# Advancing plant leaf disease detection integrating machine learning and deep learning

**DOI:** 10.1038/s41598-024-72197-2

**Published:** 2025-04-04

**Authors:** R. Sujatha, Sushil Krishnan, Jyotir Moy Chatterjee, Amir H. Gandomi

**Affiliations:** 1 School of Computer Science Engineering and Information Systems (SCORE), Vellore Institute of Technology, Vellore, India; 2 School of Computer Science and Engineering (SCOPE), Vellore Institute of Technology, Vellore, India; 3 Department of CSE, Graphic Era University, Dehradun, India; 4 Faculty of Engineering and IT, University of Technology Sydney, Ultimo, NSW 2007 Australia; 5 University Research and Innovation Center (EKIK), Óbuda University, Budapest, 1034 Hungary; 6 Department of Computer Science, Khazar University, Baku, Azerbaijan

**Keywords:** Deep learning (DL), Machine learning (ML), Plant leaf disease detection, Convolutional neural networks (CNNs), Feature extraction, Classification, Pythagoras tree, Plant sciences, Computational science, Computer science

## Abstract

Conventional techniques for identifying plant leaf diseases can be labor-intensive and complicated. This research uses artificial intelligence (AI) to propose an automated solution that improves plant disease detection accuracy to overcome the difficulty of the conventional methods. Our proposed method uses deep learning (DL) to extract features from photos of plant leaves and machine learning (ML) for further processing. To capture complex illness patterns, convolutional neural networks (CNNs) such as VGG19 and Inception v3 are utilized. Four distinct datasets—Banana Leaf, Custard Apple Leaf and Fruit, Fig Leaf, and Potato Leaf—were used in this investigation. The experimental results we received are as follows: for the Banana Leaf dataset, the combination of **Inception v3 with SVM** proved good with an Accuracy of 91.9%, Precision of 92.2%, Recall of 91.9%, F1 score of 91.6%, AUC of 99.6% and MCC of 90.4%, FFor the Custard Apple Leaf and Fruit dataset, the combination of **VGG19 with kNN** with an Accuracy of 99.1%, Precision of 99.1%, Recall of 99.1%, F1 score of 99.1%, AUC of 99.1%, and MCC of 99%, and for the Fig Leaf dataset with Accuracy of 86.5%, Precision of 86.5%, Recall of 86.5%, F1 score of 86.5%, AUC of 93.3%, and MCC of 72.2%. The Potato Leaf dataset displayed the best performance with **Inception v3 + SVM** by an Accuracy of 62.6%, Precision of 63%, Recall of 62.6%, F1 score of 62.1%, AUC of 89%, and MCC of 54.2%. Our findings explored the versatility of the amalgamation of ML and DL techniques while providing valuable references for practitioners seeking tailored solutions for specific plant diseases.

## Introduction

Machine Learning (ML) and Deep Learning (DL) have appeared as significant tools in the areas of picture classification and analysis, influencing substantial improvements in plant pathology and agriculture. Recently the utilization of these advanced technologies has collected awareness for plant leaf disease detection, advancing innovative approaches to boost productivity and crop health^1^. This paper centers on plant leaf disease detection, explicitly in fig, banana, custard apple, and potato, employing advanced techniques in DL and ML. The agriculture domain has reaped huge benefits from the synergy of ML and DL, especially in disease management and detection. Complex computational methods are used in this paradigm to interpret and evaluate complicated patterns and trends within plant disease images. It focuses on effectively employing the capabilities of DL and ML to detect and classify plant leaf images, it performs very effectively compared to conventional approaches^2^. Plant diseases that impact quality, quantity, and sustainability constantly put the global food supply system under strain. It takes a long time and is laborious to identify diseases using the traditional technique, which involves specialists. When DL and ML are introduced, the area is altered because they provide data-driven and logical approaches to fighting illnesses^3^. Because ML can recognize patterns, it is a useful technology that helps identify plant diseases automatically. Large datasets including images of plant leaves aid ML algorithms in identifying the subtle characteristics linked to different illnesses^4^. Segregated knowledge could be applied to new images, making it a reliable and suitable platform to identify diseases across different plants. DL serves as a subset of ML that outperforms in image analysis tasks. CNN important part of DL, capable of getting the hierarchical attributes from the image that ensures the better identification of the leaf disease. Such DL capabilities also enable the models to recognize intricate patterns and relations between the patterns in leaf pictures, increasing classification specificity. Despite the present potential, ML and DL applications in plant disease identification also have multiple limitations. These are the requirement for diverse and well-annotated tested samples, lack of attention to the interpretability of complex DL models, and complicated attempts to integrate these new technologies with the existing ones in agricultural practice. Still, the possibilities of increasing detection accuracy and reducing the necessity for manual inspection and crop health improvement outweigh the disadvantages^5^. Key Challenges in detecting diseases in leaves are as follows:

- Leaf Morphological Variations
- Intraspecies Disease Variations
- Multiclass Classification
- Limited Annotated Data
- User-Friendly Implementation
- Timely Detection and Intervention
- Adaptability to New Pathogens
- Integration with Agricultural Practices

Overcoming such daunting challenges may necessitate the collaboration of researchers, agriculturists, and technology developers working in the agricultural domain. However, customizing solutions based on the unique needs and eccentricities of the potatoes & figs, bananas, and custard apples is vital for successfully deploying disease detection technologies in actual agricultural areas. By facilitating DL and ML adoption in plant leaf disease detection, the present investigation takes an innovative approach to some of the issues currently affecting agriculture. More specifically, this study analyses bananas, figs, potato crops, and custard apples due to the potential dangers associated with their identification, which has a devastating effect on crop growth and food security.

The goal of this work is to investigate and clarify the developments in ML and DL methods for diagnosing illnesses in plant leaf photos. Through exploring the methods, obstacles, and innovations in this area, the study aims to support the creation of reliable and expandable solutions that have the potential to completely transform agricultural disease control techniques. We will explore the nuances of ML and DL applications in the following sections, offering an understanding of their revolutionary influence on plant pathology going forward. The remaining part of the manuscript is organized as: Sect “Literature review” presents the existing works in the areas of disease identification of plant leaves. Sect “Material and methods” presents the details of the methods and approaches followed in this paper. Sect “Results” presents the experimentation details, followed by Sect “Conclusion” offers the outcomes with a detailed argument, and Sect. 7 completes the manuscript with conceivable future exertion.

## Literature review

Ref^3^ explored plant ailment detection utilizing ML and DL methods. In the comparison, DL methods, particularly VGG-16, outperform ML methods, achieving the highest disease classification accuracy at 89.5%, showcasing the efficiency of DL in citrus plant illness findings^6^ highlighted the significance of disease detection in agriculture, specifically focusing on banana plants. It proposes an automatic method using picture segmentation for categorizing banana leaf diseases, aiming to reduce labor and detect symptoms early. Their study employs a hybrid fuzzy C-means technique for segmentation and classification, extracting color, shape, and texture characteristics. They have compared their method with existing DL approaches using quantitative metrics for diseases like black Sigatoka, yellow Sigatoka, dried/old leaves, banana bacterial wilt, and healthy plants^7^. introduced advanced image processing algorithms for early disease identification in banana leaves. The proposed CRNN–RCNN classifier achieves a high accuracy of 98%, surpassing CNN, deep CNN (DCNN), k-nearest neighbor (kNN), and support vector machine (SVM) on a banana dataset. The precision, recall, and sensitivity scores are 97.7, 97.7, and 98.69%, respectively.

Ref^8^ introduced the BananaLSD dataset, consisting of 937 original and 1600 augmented images of banana leaves affected by Sigatoka, Cordana, and Pestalotiopsis diseases. Utilized for creating the BananaSqueezeNet model, the dataset was captured using smartphone cameras in diverse real-world conditions. It holds potential for ML models facilitating early symptom identification by farmers & aids as a treasured reserve for investigators studying leaf spot diseases^9^. proposed a novel DL technique, ACO-CNN, for plant leaf disease detection. Leveraging ant colony optimization (ACO) and a CNN classifier, it extracts color, texture & leaf procedure geometries from leaf images. The method outperforms existing techniques, as indicated by superior accuracy rates and effectiveness metrics. The disease detection process involves picture acquisition, image separation, noise removal, and classification^10^. introduced a framework for plant disease recognition, leveraging DL and traditional features. The method used a deep feature signifier utilizing transfer learning and feature fusion to capture local texture data in plant leaf pictures. Center loss is combined to boost discriminatory features. The proposed approach achieves high classification accuracies of 99.79, 92.59 & 97.12% on three datasets (2 Apple Leaf and 1 Coffee Leaf), demonstrating its effectiveness in distinguishing plant leaf diseases^11^. emphasized the need for timely recognition & categorizing of potato leaf illnesses in agronomy. Their proposed technique, using an improved DL algorithm, classifies potato leaves into five classes, addressing limitations in existing methods. The model achieves efficient classification, employing a pre-trained Efficient DenseNet with additional features. A reweighted cross-entropy loss function handled data imbalance, and dense connections by regularization diminish overfitting. The algorithm, a novel approach, successfully perceives & categorizes four ailments in potato leaves, demonstrating an accuracy of 97.2%. Their experimental results indicated its superiority over existing models.

Ref^12^ discussed disease prevention in Indonesian potato production using the Swin Transformer, a highly efficient and accurate DL model.

**Fig.1:**
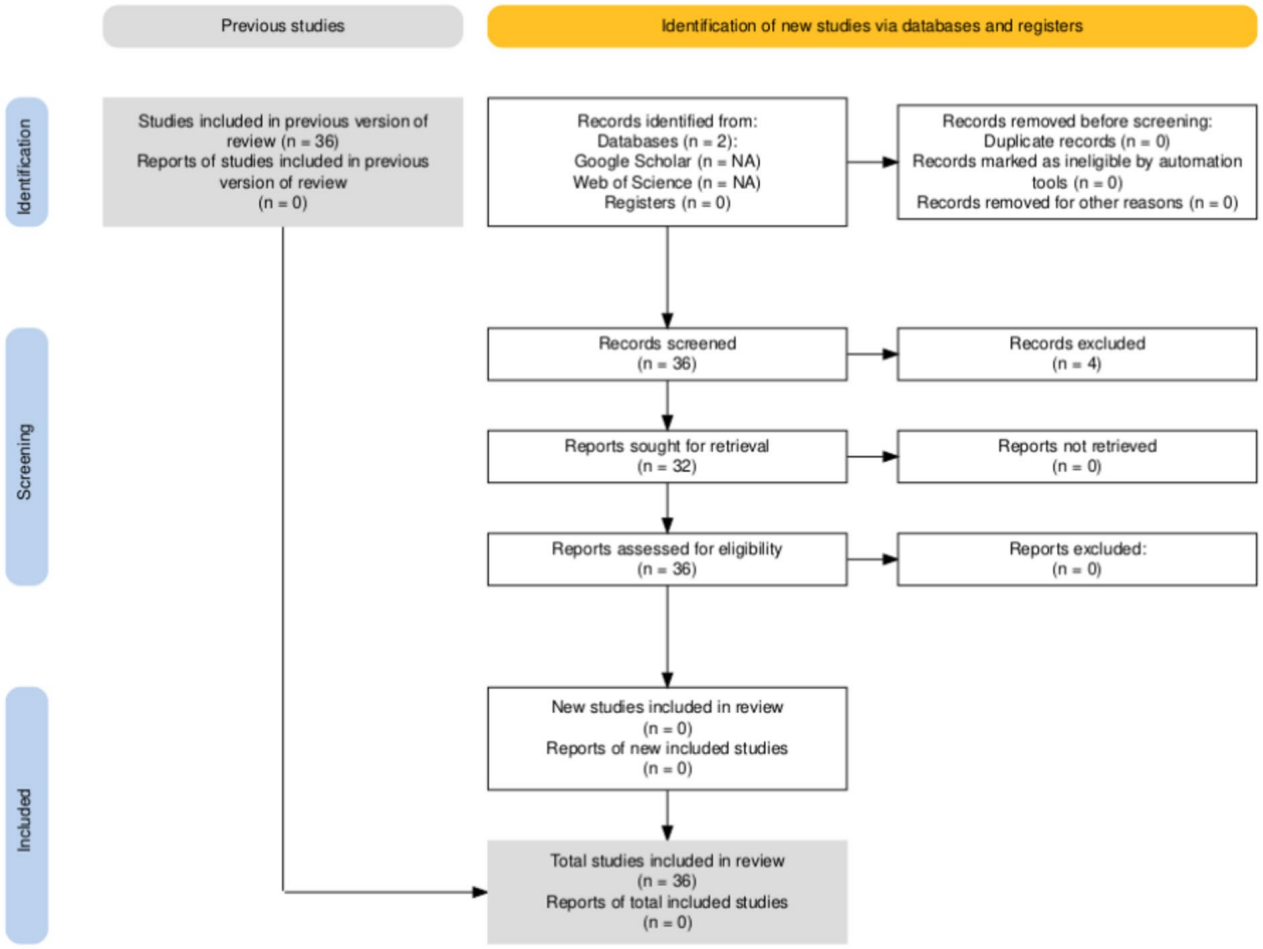
PRISMA flow diagram for systematic literature review.

Swin Transformer have shown promising results in detecting diseases in potato leaf with an accuracy of 97.70% suggesting its possible breakthrough in agricultural research. Detecting leaf disease of crops was covered in^13^ utilizing the DL architectures VGG-19, Inception v3, VGG-16, and DenseNet-121. DenseNet-121 outperformed other models with the highest accuracy of 91.75%, addressing the challenges in manual identification with CNN-based fruit disease classification^14^. MobileNet v2, VGG-16, and DenseNet-121 have shown higher accuracy and recall in predicting the disease of papaya, guava, and citrus respectively^15^. has observed that the VGG-16-based model produces a remarkable accuracy of 99.7% and AUC of 93.3% for classifying nine different tomato diseases of the PlantVillage dataset^16^. devised a strategy for evaluating custard apple leaves for disease and NPK deficiency detection. The system was able to achieve an accuracy of 99.5% by employing supervised ML techniques like SVM and kNN. This system enables us to efficiently monitor plant health. In custard apple trees identification and treatment of diseases play a crucial role as if untreated it can lead to damage and decreased agricultural productivity. Expert systems trained to diagnose and suggest remedies can assist farmers in administering precise treatment^17^. Ref^18^ emphasized the pivotal role of image processing and ML in precise disease diagnosis for the custard apple plant, they have also focused on the analysis of leaf parameters. Ref^19^ has culminated the issues faced and given a walkthrough of difficulties and solutions for data collection, segmentation, and classification^20^. employed ultrasonic techniques and ML to detect defects in standing trees based on holes. The study has shown promising results in both lab and field study, with a one-dimensional CNN and fine Gaussian support vector machine working effectively^21^. proposed an Internet of Things and DL-based irrigation system called DLiSA, to optimize water utilization across various climatic conditions. It outperforms existing models, highlighting its performance in experimental farming scenarios^22^. developed a ML-based crop growth and disease monitor and provided real-time suggestions to the farmers. It utilized ensemble classification and pattern recognition. According to their study ensemble nonlinear SVM outperformed other ML methods.(Fig 1)

A tabular comparison is presented in Table 1.

**Table 1:** Comparative analysis.

Sl. No	Reference	Dataset used	Algorithms	Disease detection/identification	Description/outcome
1	^16^	Custard Apple Leaf diseases	kNN and SVM	Leaf parameter analysis, detection of N, P, K deficiencies, and leaf diseases	Accuracy-99.5%
2	^3^	Citrus Leaf Disease	SVM, SGD, RF, Inceptionv3, VGG16, VGG19	Canker, Blackspot, Greening, Melanose, Healthy	Classification Accuracy of RF-76.8%, SGD-86.5%, SVM-87%, VGG19-87.4%, Inceptionv3-89%, VGG16-89.5%
3	^17^	Custard Apple disease	Expert System	Anthracnose, Leaf spot, Diplodia rot, Black canker, Spiral nematode, and Stunt nematode, IPM for Custard Apple	Expert System Developed
4	^10^	2 Apple Leaf and 1 Coffee Leaf	DL	A general framework for recognizing plant diseases	The proposed method achieves 99.79%, 92.59%, and 97.12% classification accuracies on the three datasets
5	^6^	Alliance of Bioversity International and CIAT Banana Image Library	Total generalized variation fuzzy C means, CNN	Disease Classification	Sesitivity-89.04%, Specificity-96.38%, and Accuracy- 93.45%
6	^7^	Banana Plant Leaf Disease	Histogram pixel localization, region-based edge normalization, Gabor-based binary patterns, convolution RNN, Convolutional Recurrent Neural Network–Region-Based Convolutional Neural Network (CRNN–RCNN)	Disease Classification	Precision-97.7%, recall-97.7%, sensitivity-98.69%, accuracy-98%
7	^15^	PlantVillage	VGG16-based model	Disease Classification into 9 major types	Accuracy-99.7% and area under the curve (AUC)-93.3%
8	^9^	Citrus fruits and leaves dataset	Ant Colony Optimization with Convolution Neural Network (ACO-CNN)	Classification of leaves	Accuracy-99.98%
9	^12^	Potato Leaf Disease	Swin Transformer DL	Classification of healthy and unhealthy leaves	Accuracy(training)-99.70%
10	^11^	PlantVillage	Modified DenseNet-201	Potato Late Blight (PLB), Potato Early Blight (PEB), Potato Leaf Roll (PLR), Potato Verticillium_wilt (PVw) and Potato Healthy (PH) class	Accuracy-97.2%
11	^14^	Papaya Fruit Diseases, Guava Leaves and Fruits Dataset, Citrus Fruits and Leaves Dataset	MobileNet-v2, VGG16, DenseNet121	Detect disease in fruits via images	MobileNetv2 model, the disease prediction accuracy for papaya, guava, and citrus was 99.4%, 98.8%, and 95.8% and the recall values were 99.4%, 98.8%, and 93.8%, respectively.VGG16, the disease prediction accuracy for papaya, guava, and citrus was 97.7%, 99.6%, and 94.2% and the recall values were 96.5%, 99.6%, and 89.2%, respectively.DenseNet121, the disease prediction accuracy for papaya, guava, and citrus was 99.4%, 97.6%, and 99.2%, and the recall values were 98.8%, 97.6%, and 99.2%, respectively
12	^13^	–	VGG-16, VGG-19, InceptionV3, and DenseNet-121	Leaf Disease Identification	Accuracy-91.75% on DenseNet-121

The literature evaluation concludes that the use of DL models, including VGG-19 and Inception v3, in conjunction with ML approaches greatly improves the precision and efficacy of plant disease identification. AI has the potential to completely transform agricultural disease control, as seen by various automated approaches' superior performance over labor-intensive traditional techniques. The adaptability and robustness of the combined ML and DL methodology are highlighted by the models’ high performance and versatility across a variety of datasets, such as potato, banana, custard apple, and fig leaves. These insights can be used to develop customized solutions for particular plant diseases.

## Material and methods

**Fig.2:**
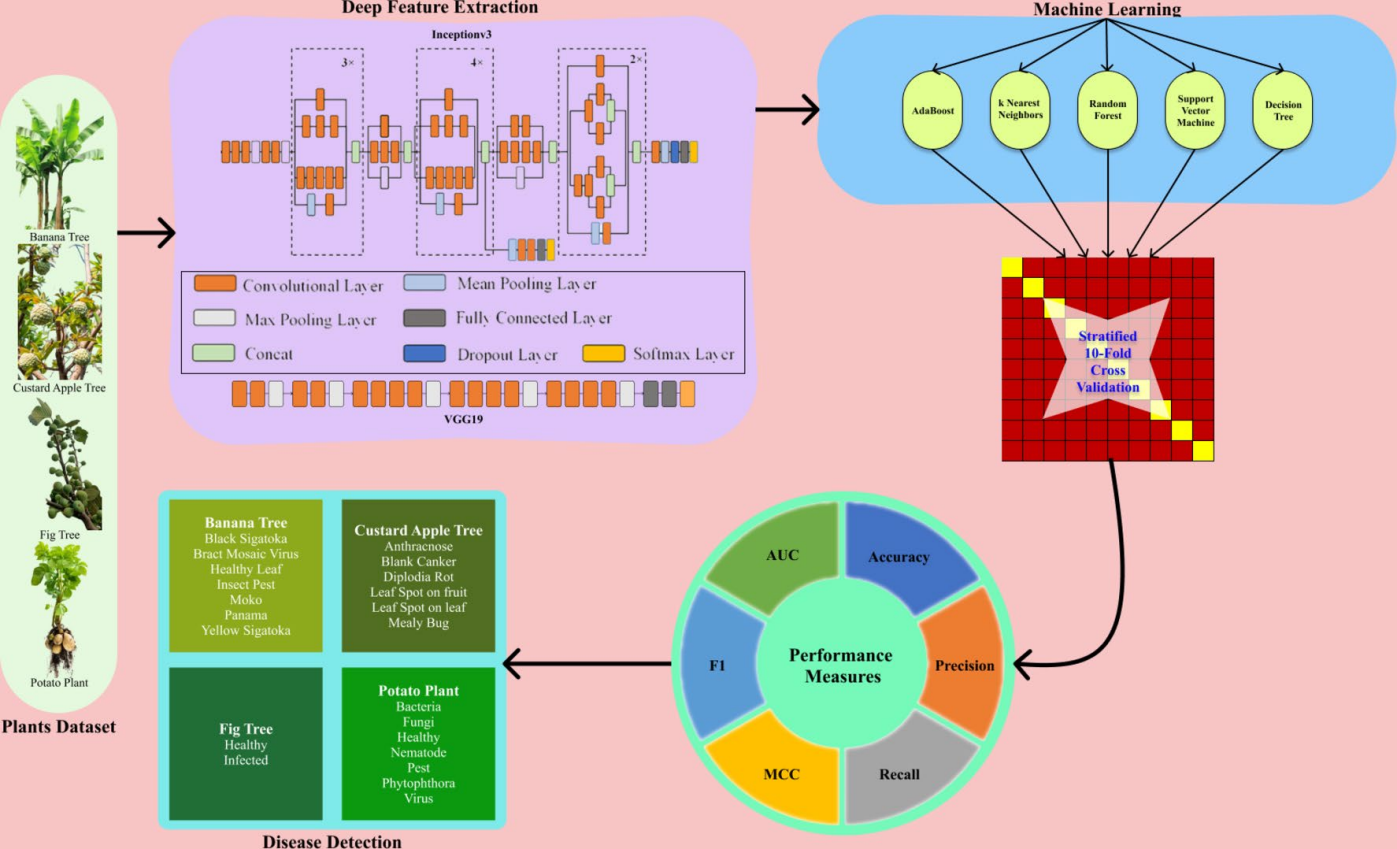
Fusion of DL and ML for Plant Leaf Disease Detection.

Figure 2 presents the detailed work of the proposed approach.

### Dataset

In the present work, we have used 4 datasets^23–26^. Ref^23^ includes 408 original banana images from real fields of Dhaka, Bangladesh. The dataset is distributed in 7 classes namely: Black Sigatoka—67 images, Bract Mosaic Virus – 50 images, Healthy Leaf – 86 images, Insect Pest – 86 images, Moko – 55 images, Panama – 41 images, Yellow Sigatoka – 23 images respectively. Ref^24^ compiled a dataset comprising 8226 pictures showcasing various diseases affecting Sugar Apples/Custard Apples from Pune, India, encompassing Anthracnose, Blank Canker, Diplodia Rot, Leaf Spot on fruit, Leaf Spot on leaf, and Mealy Bug. The dataset is distributed in 6 classes namely: Anthracnose – 1075 images, Blank Canker – 1780 images, Diplodia Rot – 1645 images, Leaf spot on leaves – 1255, Leaf spot on fruit—867 images, Mealy Bug – 1604 images respectively^25^ provides a dataset comprising 2321 images of Fig leaves, distinguishing between 1350 infected with the fig leafworm and 971 healthy ones from different regions of Iraq. The dataset is intended for research on the impact of fig leafworm infestation on production. The dataset is distributed in 2 classes namely: Healthy – 971 images and Infected – 1350 images respectively^26^. provides a dataset with 3076 images of 1500 × 1500 pixels representing different classes of potato leaf diseases caused by fungi, viruses, pests, bacteria, Phytophthora, nematodes, as well as healthy leaves from Central Java, Indonesia. The dataset is distributed in 7 classes namely: Bacteria – 569 images, Fungi – 748 images, Healthy – 201 images, Nematode – 68 images, Pest – 611 images, Phytophthora – 347 images, Virus – 532 images respectively.

### System components

#### Deep feature extraction

Pre-trained models help in effective feature extraction and, in turn, provide perfect classification. Plant leaf datasets from the benchmarked repository are cumulated and efficiently handled to build disease detection. Performance measures of various combinations of deep and ML algorithms utilized.

##### Inception v3

The Inception v3 model was developed based on the inceptionv1, and it is enhanced and outperforming its precursor. Multiple strategies were incorporated in Inception v3 to optimize the model’s network for better adaptability and higher efficiency. It is faster and computationally less expensive than its predecessors inceptionv1 and Inception v3, though it has a larger network compared with them. Auxiliary Classifiers are being used as regularizers. The pivotal changes implemented in Inception v3 are making convolutional factorizations into smaller convolutions, making asymmetric convolutions from spatial factorization, employing auxiliary classifiers, and reducing the grid size to a considerable extent^27^.

##### VGG19

VGG19 is named for its 19 weighted layers, comprising 16 convolutional layers, five Max Pooling layers, and three Dense layers, totaling 21 layers. However, only sixteen of these layers are weight layers or learnable parameters layers. The input tensor size for VGG19 is 224 by 224, with three RGB channels. Notably, VGG19 is characterized by its emphasis on 3 × 3 filter convolution layers with a stride of 1, prioritizing simplicity over an abundance of hyper-parameters. The architecture consistently employs the same padding and a max pool layer with a 2 × 2 filter and stride 2. The arrangement of convolution and max pool layers is uniform throughout the entire architecture. The Conv-1 Layer has 64 filters, Conv-2 has 128 filters, Conv-3 has 256 filters, and Conv-4 and Conv-5 each have 512 filters. Following the stack of convolutional layers are three fully connected (FC) layers, with the third performing a 1000-way ILSVRC classification with 1000 channels. Each of the first 2 FC layers has 4096 channels. The last soft-max layer is configured to the class of the plant dataset^28^.

#### Machine learning (ML)

The evolution of the decision-making process without coding is a breakthrough in the data handling domain. Based on the huge data, recognizing the pattern, and forecasting the results are accomplished with the help of ML algorithms. Separate codes are not required due to the advancement in the ML area that in turn helps in making vital decisions. The usage of ML in analyzing and interpreting is embedded in all the applications from small to big in all the domains from physical science to health science to disclose the patterns that exhibit certain illnesses^29^.

Active ML work is carried out persistently by professionals and researchers to improve the efficiency of recognizing the ailments of a budding sage, forecasting the same to make better decisions on providing the matching treatment. In illness detection, a prompt strategy to make precise diagnosing and enhancing will be achieved by employing the ML process.

##### AdaBoost

It falls under the ensemble learning (EL) approach, where repeatedly cumulates the weak learners, give the misclassified cases a bigger weight. On the whole, the model puts a high level of importance on the phase of accurate classification and nurtures the methodology to upsurge the accuracy. Precisely, the weighted sum of these learners. It shows its brilliance in the case of binary classification^30^.

##### k-nearest neighbors (k-NN)

Normally provides great support both in the case of regression and classification problems in the ML domain. Various types of distance metrics help in labeling the instances based on the arrangement of the data in the feature space and the closest will be mapped. In the case of the huge dataset, the cost involved in the computation is high and acts as a challenge to proceed even though it is easy and non-parametric. To get maximum efficiency, the selection of the correct K value is highly important and crucial^31^.

##### Random forest (RF)

It is a popular EL approach that utilizes randomized attributes and data subsets to produce many DTs. The process of voting in classification or averaging in regression and integrating tree predictions, reduces the overfitting and optimizes the generalization. It is honored for performing well in a range of situations in exhibiting nice precision and flexibility^3^.

##### Support vector machine (SVM)

The task concentrates on finding the best hyperplane to optimize the margins among the support vectors in the process of segregating across the classes. For non-linear patterns, the kernel approach is recommended. It performs well in the high-dimensional areas even though it has high sensitivity in case of outliers. The C parameters balance the perfect categorization and even boundary decision. SVM is highly prescribed for its adaptability and efficiency both in the case of regression and classification problems of supervised ML^32^.

##### Decision tree (DT)

The ML algorithm is suited more for regression and classification problems. It hierarchically arranges the information into a tree and at each node, decisions will be taken based on its properties. It suits the ensemble technique too and is prone to highly interpretable and overfitting nature. The main pros are their malleability and simple structure^33^.

## Results

### Stratified 10-Fold cross validation

It's a unique approach in the process of evaluation in the ML models. In this process, the entire dataset is subdivided into 10 subgroups, ensuring the class spreading of the original data is sustained in each subgroup. Over the model, 10 iterations of training and testing are performed. In each iteration, the distinct testing subset is maintained. It serves more perfectly in the case of unequal distribution of the class in the data^3^.

### Confusion matrix (CM)

In the case of any class situation, CM measures are used often to estimate and take appropriate measures to curtail classification problems. The CM illustrates the matrix between predicted and actual values. True Negative precisely named TN, represents the perfectly predicted negative classes. Similarly True Positive stands for TP, and references perfectly predicted positive classes. False Positive or FP, denotes the real negative instances erroneously mapped as positive. Conversely, “FN” (False Negative) refers to instances in which true positive examples were mistakenly labeled as negative^32^.

### Accuracy

Every match, which contrasts the actual and predicted classes of every data point, indicates one accurate prediction in terms of accuracy. Next, the number of correct predictions divided by the total number of forecasts is used to determine accuracy^14^. The equation is:

1 $$Accuracy \, = \, TP \, + \, TN \, / \, \left( {TP \, + \, TN \, + \, FP \, + \, FN} \right)$$

### Precision

Precision is defined as the ratio of correctly categorized positive samples (True Positive) to the total number of positively classified samples (it can be either correct or erroneous)^14^. The equation is:

2 $$Precision = TP \, / \, TP + FP$$

### Recall

The fraction of Positive samples that were correctly anticipated to be Positive to all Positive samples is how the recall is calculated. The recall measures the model’s ability to recognize positive samples. The recall increases with the number of positive samples found^14^. The equation is:

3 $$Recall = TP/ \, TP + FN$$

### F1-score

The F1-score combines evaluations of a model's recall and accuracy. The accuracy statistic counts the number of times a model correctly predicted a given dataset. Overall performance is good for a classification model with a high F1 score. It demonstrates how the model can effectively detect positive situations while minimizing FP and FN^34^.

### Area under curve (AUC)

This metric is used to assess how well a classification model predicts binary outcomes. It is often used in ML and data analytics. The True Positive Rate (TPR) vs False Positive Rate (FPR) at different thresholds are plotted, and the area under the resultant curve is calculated to find the AUC^14^.

### Matthew’s correlation coefficient (MCC)

The best single-value classification metric for condensing an error or confusion matrix is MCC^35^. Moreover, MCC also varies between + 1 and −1 to accommodate the majority of correlation coefficients as:

- The best agreement between the actual and anticipated values is + 1.
- 0 denotes no consensus. That is, based on the facts, predictions are arbitrary.
  4 $$MCC \, = \, \left( {TN{\text{ x }}TP \, {-} \, FN{\text{ x }}FP} \right) \, / \, SQRT\left( {\left( {TP \, + \, FP} \right) \, \left( {TP \, + \, FN} \right) \, \left( {TN \, + \, FP} \right) \, \left( {TN \, + \, FN} \right)} \right)$$

### Pythagorean tree (PT)

PT answers questions about the class succinctly. The number of training cases covered by the size of the squares on nodes in Pythagorean trees^36^. The corresponding new squares form a right triangle on top of the parent square once the data is divided into two subsets^37^.

## Discussion

The accuracy of the classifiers in predicting disease categories from the plant dataset was examined in this work. The outcomes of these analyses are displayed. To gauge the accuracy of the integration of deep and ML in the identification of plant disease processes, several performance measures were employed. Table 2 provides insight into performance measures in the case of banana plants. The blend of Inception v3 and SVM shows higher values in all performance measures and has a higher accuracy of 91.9%. The disease detection will be reliable in the case of Inception v3 along with SVM.

**Table 2:** Banana plant leaf- performance measures.

DL Approach	ML Methods	Accuracy	Precision	Recall	F1	AUC	MCC
***Inception v3***	AdaBoost	0.598	0.606	0.598	0.601	0.761	0.522
	kNN	0.88	0.878	0.88	0.878	0.983	0.857
	RF	0.762	0.762	0.762	0.756	0.947	0.715
	***SVM***	***0.919***	***0.922***	***0.919***	***0.916***	***0.996***	***0.904***
	DT	0.654	0.666	0.654	0.658	0.803	0.59
VGG19	AdaBoost	0.706	0.7	0.706	0.705	0.825	0.649
	kNN	0.841	0.846	0.841	0.836	0.967	0.811
	RF	0.806	0.804	0.806	0.8	0.963	0.769
	SVM	0.892	0.895	0.892	0.89	0.987	0.872
	DT	0.721	0.72	0.721	0.719	0.841	0.666

Significant values are in bold.

**Fig.3:**
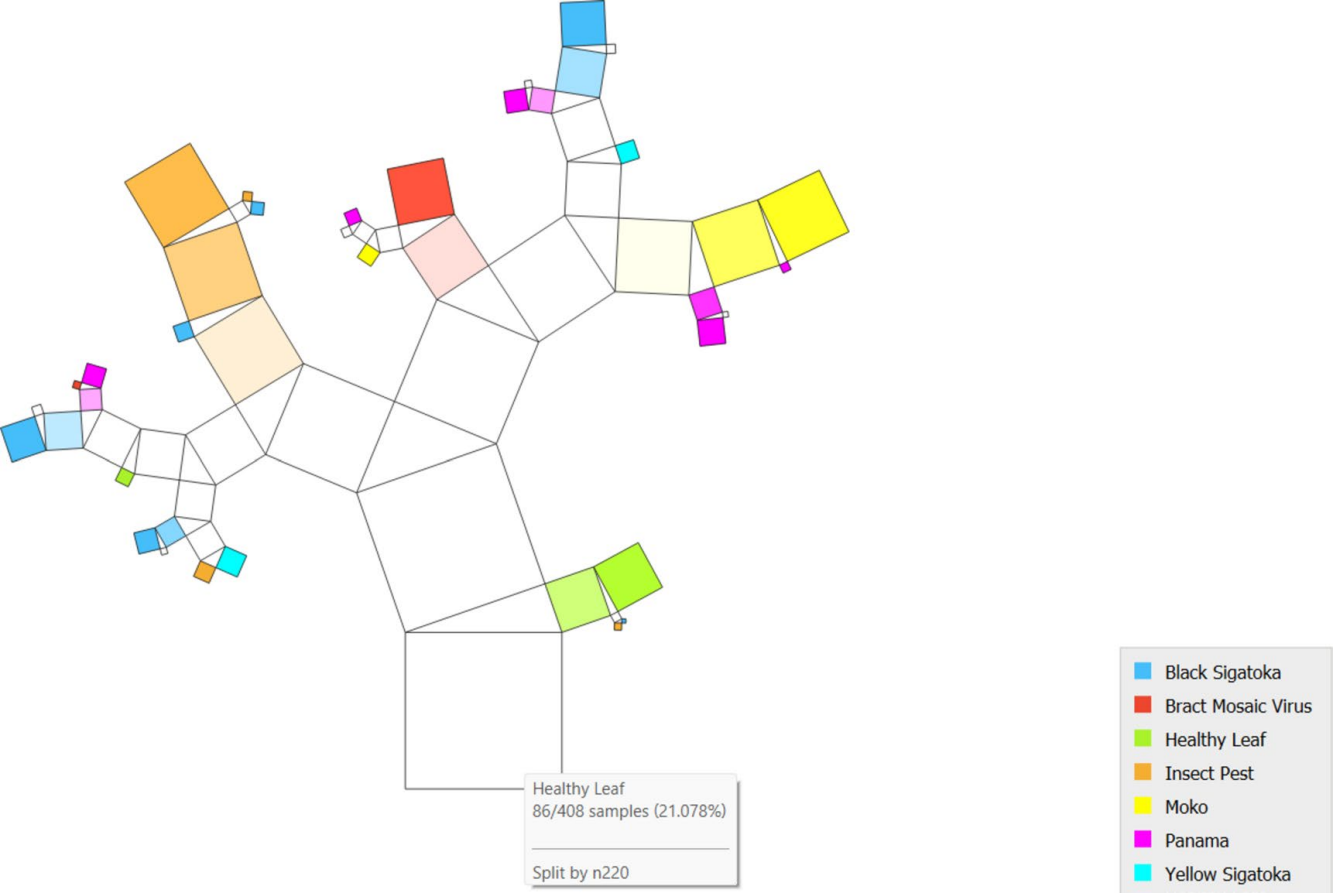
PT – Inception v3 + DT.

Figure 3PT via the Inception v3 approach along with DT illustrates the various classes based on the feature distribution. It is evident in the case of the banana leaf, which holds 7 classes, and 408 records are classified into different combinations.

**Fig.4:**
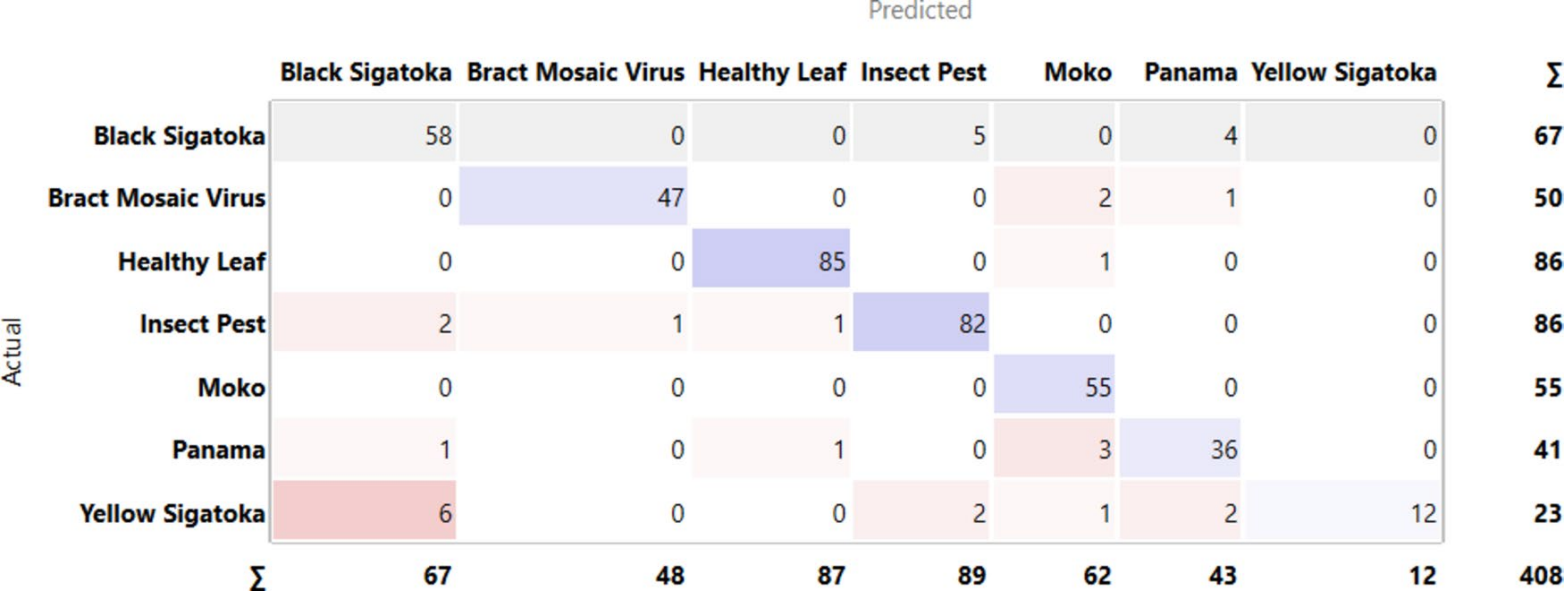
CM– Inception v3 + SVM.

Figure 4 indicates the diagonal values are correctly classified records and either side of the diagonals represents misclassified records. In the case of black Sigatoka 58 images are correctly predicted out of 67, bract mosaic virus 47 images are correctly predicted out of 50, healthy leaf 85 are correctly predicted out of 86, insect pest 82 are correctly predicted out of 86, moko 55 are predicted correctly on whole, Panama 36 predicted perfectly out of 41 and yellow Sigatoka 12 out of 23 are predicted perfectly.

Table 3 provides insight into performance measures in the case of the custard apple. The blend of VGG19 and kNN shows higher values in all performance measures and has a higher accuracy of 99.1%. The disease detection will be reliable in the case of VGG19 along with kNN.

**Table 3:** Custard Apple Leaf and Fruit- Performance Measures.

DL Approach	ML Methods	Accuracy	Precision	Recall	F1	AUC	MCC
Inception v3	AdaBoost	0.745	0.745	0.745	0.745	0.845	0.69
	kNN	0.986	0.986	0.986	0.986	0.999	0.982
	RF	0.874	0.874	0.874	0.873	0.979	0.847
	SVM	0.858	0.865	0.858	0.856	0.981	0.83
	DT	0.756	0.756	0.756	0.756	0.835	0.704
***VGG19***	AdaBoost	0.761	0.761	0.761	0.761	0.854	0.709
	***kNN***	***0.991***	***0.991***	***0.991***	***0.991***	***0.999***	***0.99***
	RF	0.903	0.904	0.903	0.903	0.987	0.883
	SVM	0.953	0.954	0.953	0.953	0.997	0.943
	DT	0.765	0.765	0.765	0.765	0.852	0.715

Significant values are in bold.

**Fig.5:**
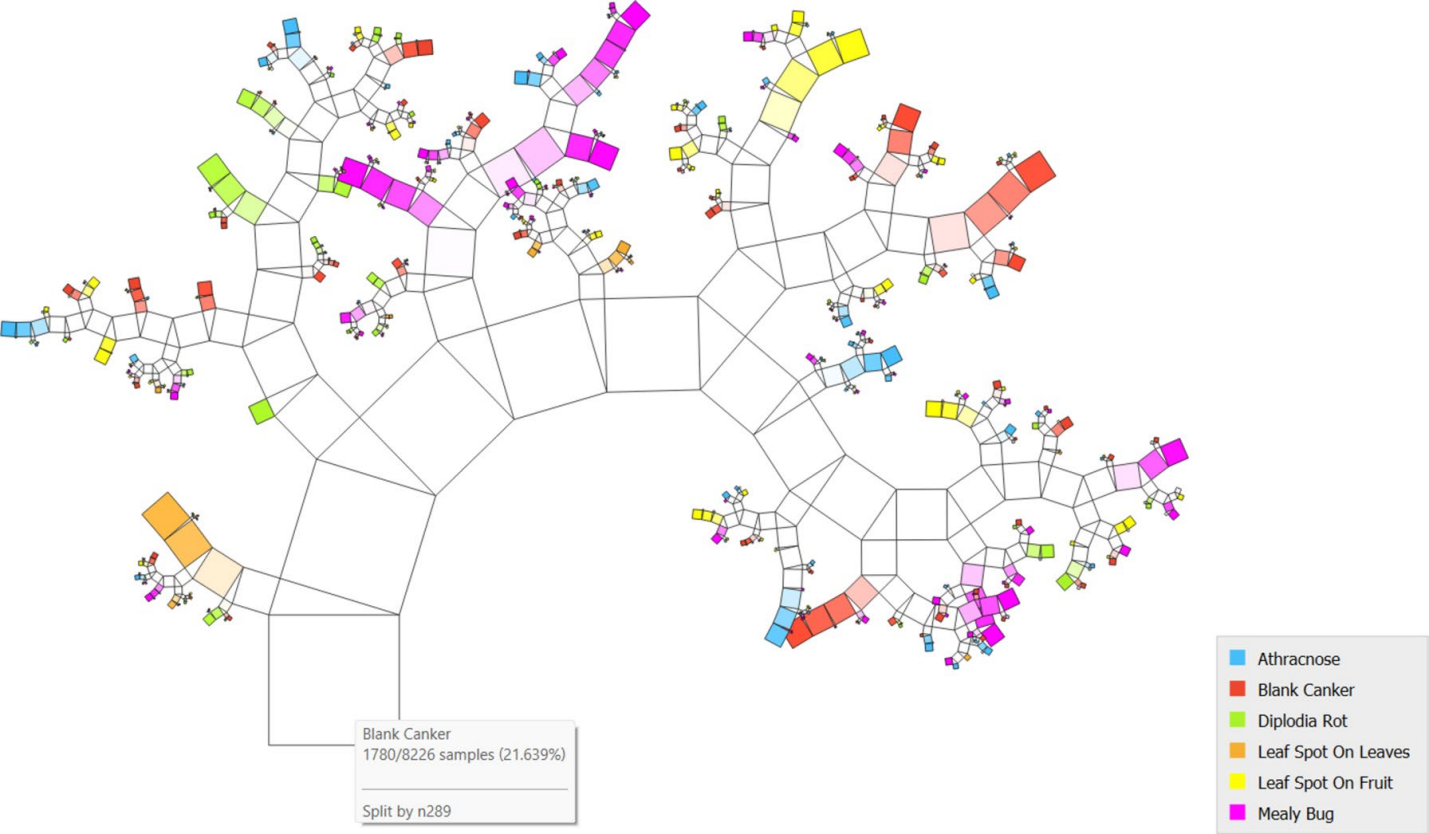
PT – VGG19 + DT.

Figure 5 PT via the VGG19 approach along with DT illustrates the various classes based on the feature distribution. It is evident in the case of the custard apple leaf and fruit, 6 classes, and shows that 8226 instances are classified over different combinations.

**Fig.6:**
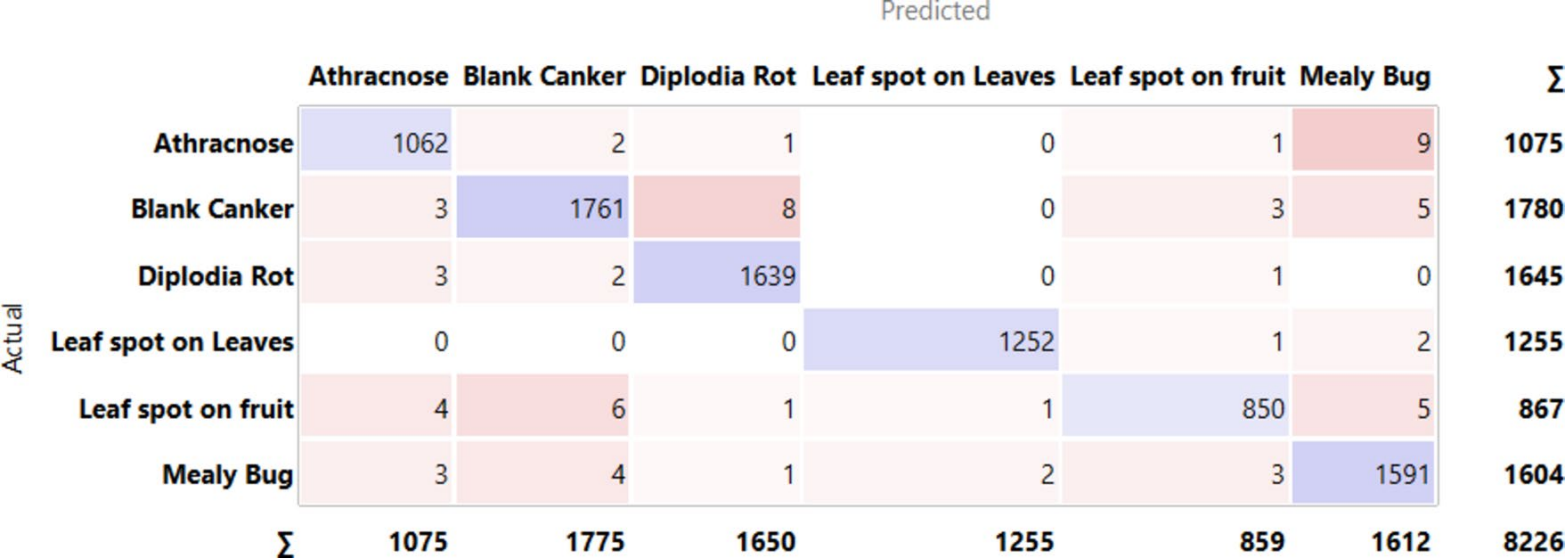
CM– VGG19 + kNN.

Figure 6 indicates the diagonal values are correctly classified records and either side of the diagonals represents misclassified records. In the case of black anthracnose, 1062 images are correctly predicted out of 1075, blank canker 1761 images are correctly predicted out of 1780, diplodia rot 1639 are correctly predicted out of 1645, leaf spot on leaves 1252 are correctly predicted out of 1255, leaf spot on fruit 850 are predicted correctly out of 876 and mealy bug 1591 predicted perfectly out of 1604.

Table 4 provides insight into performance measures in the case of Fig plant. The blend of VGG19 and kNN shows higher values in all performance measures and has a higher accuracy of 86.5%. The disease detection will be reliable in the case of VGG19 and kNN.

**Table 4:** Fig Leaf – Performace Measures.

DL Approach	ML Methods	Accuracy	Precision	Recall	F1	AUC	MCC
Inception v3	AdaBoost	0.772	0.772	0.772	0.772	0.767	0.532
	kNN	0.863	0.863	0.863	0.862	0.935	0.717
	RF	0.822	0.822	0.822	0.822	0.912	0.634
	SVM	0.848	0.848	0.848	0.847	0.922	0.686
	DT	0.79	0.789	0.79	0.79	0.723	0.567
***VGG19***	AdaBoost	0.776	0.776	0.776	0.776	0.77	0.54
	***kNN***	***0.865***	***0.865***	***0.865***	***0.865***	***0.933***	***0.722***
	RF	0.826	0.826	0.826	0.826	0.91	0.642
	SVM	0.76	0.759	0.76	0.759	0.841	0.503
	DT	0.781	0.78	0.781	0.78	0.732	0.547

Significant values are in bold.

**Fig. 7:**
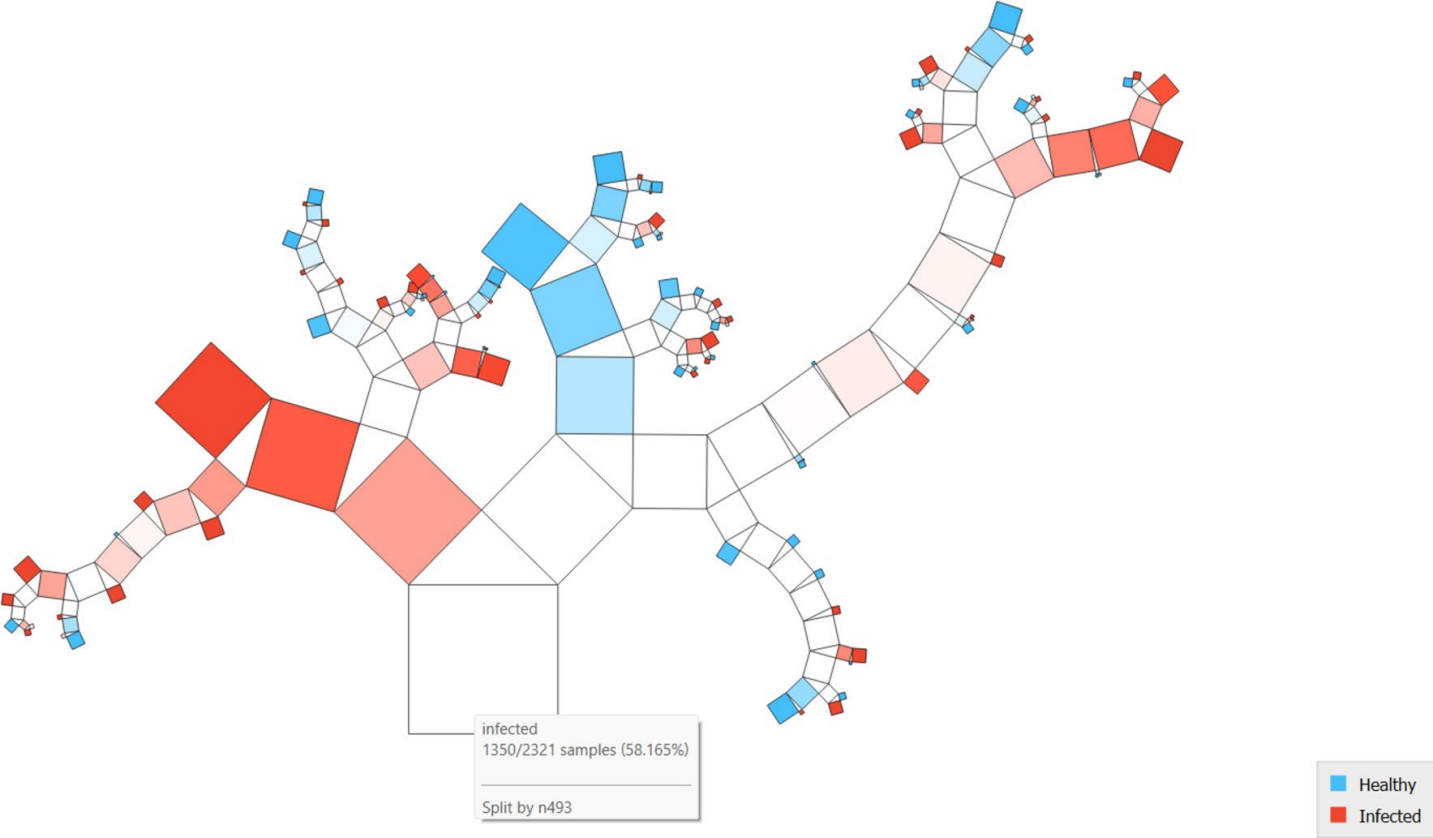
PT – VGG19 + DT.

Figure 7 PT via the VGG19 approach along with DT illustrates the various classes based on the feature distribution. It is evident in the case of the custard apple tree, 2 classes, and shows that 2321 instances are classified over different combinations.

**Fig.8:**
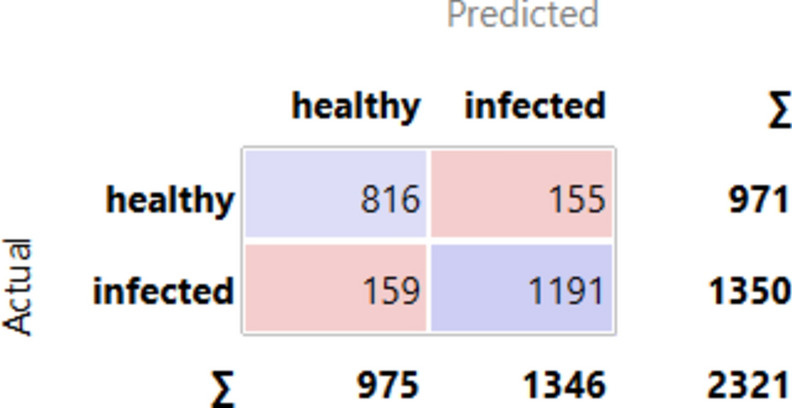
CM– VGG19 + kNN.

Figure 8 indicates the diagonal values are correctly classified records, and either side of the diagonals represents misclassified records. In the case of healthy 816 images are correctly predicted out of 971, and in infected 1191 images are correctly predicted out of 1350.

Table 5 provides insight into performance measures in the case of potato plants. The blend of Inception v3 and SVM shows higher values in all performance measures and has a higher accuracy of 62.6%. The disease detection will be reliable in the case of Inception v3 and SVM.

**Table 5:** Potato Leaf- Performance Measures.

DL Approach	ML Methods	Accuracy	Precision	Recall	F1	AUC	MCC
***Inception v3***	AdaBoost	0.569	0.589	0.569	0.556	0.843	0.476
	kNN	0.589	0.599	0.589	0.586	0.839	0.495
	RF	0.502	0.495	0.502	0.489	0.785	0.384
	***SVM***	***0.626***	***0.63***	***0.626***	***0.621***	***0.89***	***0.542***
	DT	0.425	0.426	0.425	0.425	0.651	0.298
VGG19	AdaBoost	0.607	0.623	0.607	0.599	0.86	0.515
	kNN	0.608	0.607	0.608	0.6	0.849	0.521
	RF	0.532	0.533	0.532	0.523	0.805	0.422
	SVM	0.618	0.617	0.618	0.611	0.891	0.532
	DT	0.425	0.423	0.425	0.424	0.644	0.298

Significant values are in bold.

**Fig.9:**
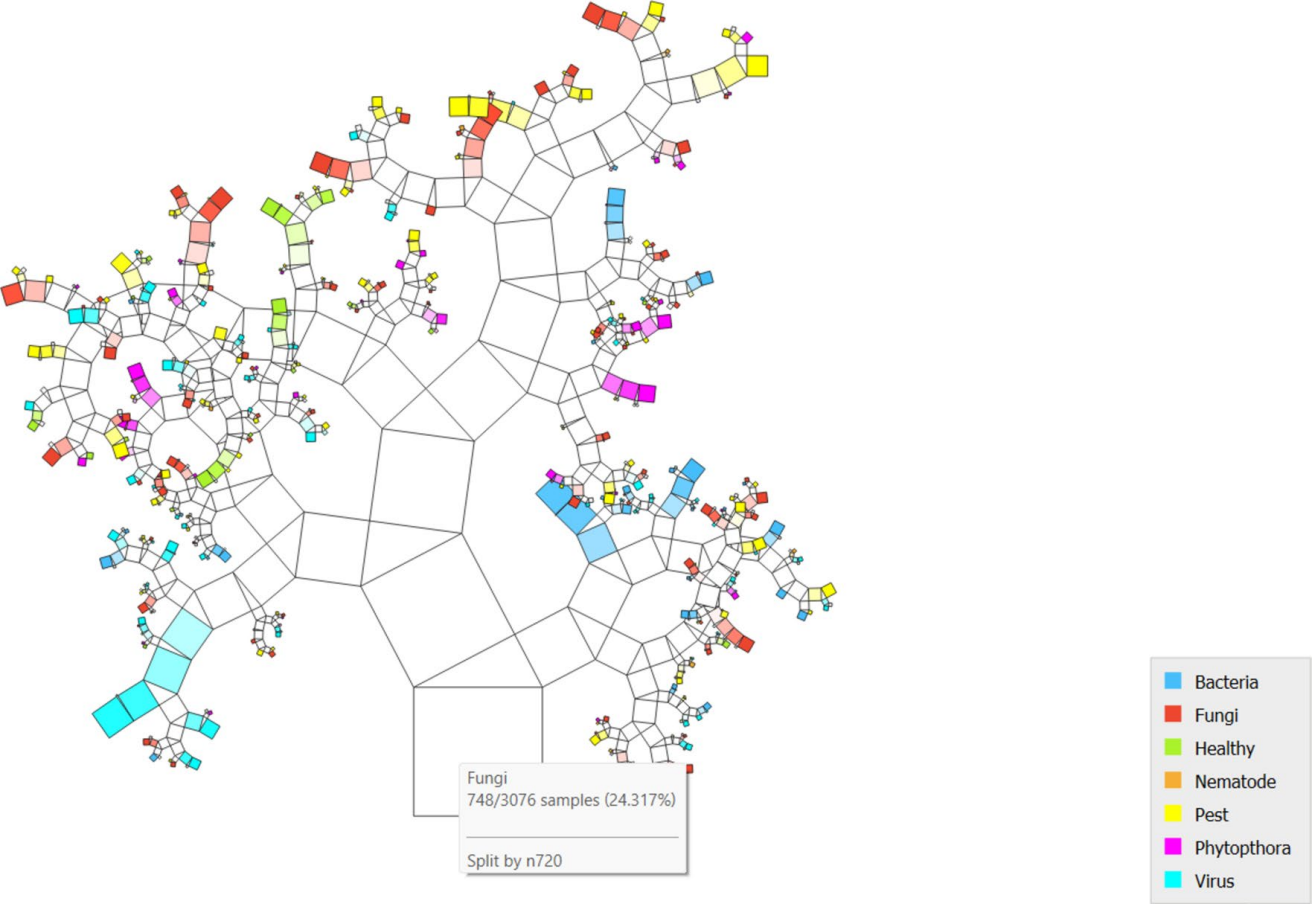
PT – Inception v3 + DT.

Figure9 PT via the Inception v3 approach along with DT illustrates the various classes based on the feature distribution. It is evident in the case of the potato leaf, 7 classes, and shows that 3076 instances are classified over different combinations.

**Fig.10:**
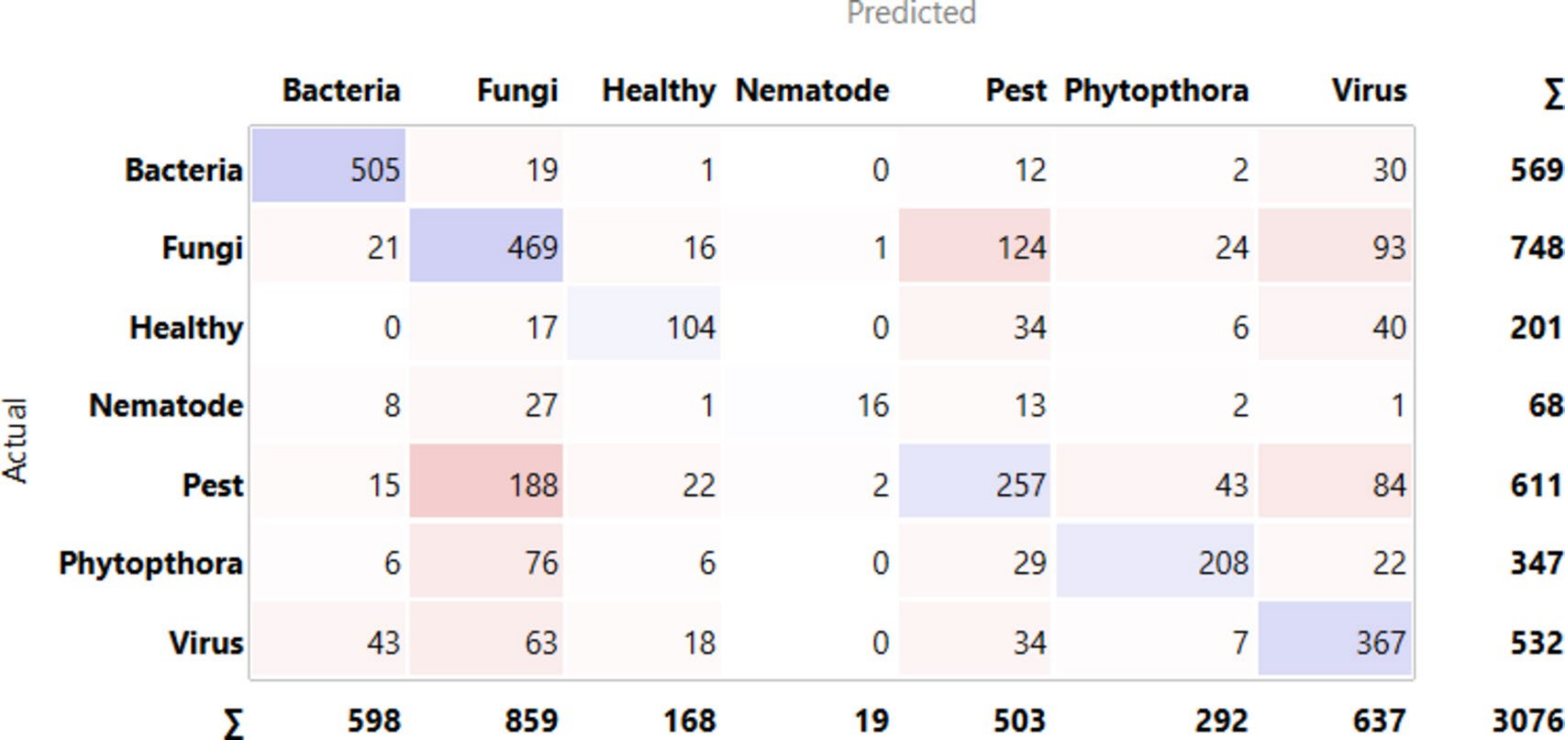
CM– Inception v3 + SVM.

Figure 10 indicates the diagonal values are correctly classified records and either side of the diagonals represents misclassified records. In the case of bacteria, 505 images are correctly predicted out of 569, fungi 469 images are correctly predicted out of 748, 104 are correctly predicted out of 201, nematode 16 are correctly predicted out of 68, pests 257 are predicted correctly out of 611, phytophthora 208 predicted perfectly out of 347 and virus 367 out of 532 are predicted nicely.

Table 6 presents the comparison of the proposed work with other existing works.

**Table 6:** Performance Comparison.

Sl. No	Reference	Approach	Accuracy
1	^38^	Hierarchical SVM classification (Citrus Leaf disease dataset)	91.76%
2	^6^	CNN with TGVFCMS (Alliance of Bioversity International and CIAT Banana Image Library dataset)	93.45%
3	^39^	Deeper lightweight convolutional neural network architecture (DLMC-Net) (PlantVillage dataset)	93.56%
4	^40^	Rehearsal-based class-incremental learning approaches (PlantVillage dataset)	66.60%
5	Proposed Method	Inception v3 with SVM (Banana dataset) VGG19 with kNN (Custard Apple Leaf and Fruit dataset) VGG19 with kNN (Fig Leaf dataset) Inception v3 with SVM (Potato Leaf dataset)	91.9% 99.1% 86.5% 62.6%

## Conclusion

In conclusion, this study presents a pioneering investigation into the synergistic fusion of DL & ML techniques for the accurate detection of plant leaf diseases, focusing on banana, fig, potato, and custard apple plants. The integration of DL for feature extraction and ML for classification proved to be a powerful approach, addressing challenges related to leaf morphological variations, disease diversity, and environmental influences. The results underscore the effectiveness of the proposed synergistic fusion approach, showcasing superior performance compared to standalone methodologies.

The proposed approach has the potential to revolutionize automated plant disease diagnostics, support sustainable farming methods, and improve crop health.

Researchers, agricultural specialists, and tech developers may learn a lot from the study's thorough analysis of issues, previous efforts, and experimental outcomes. The broad application of these technologies in agriculture depends on their capacity to handle problems like insufficient annotated data, adaptation to novel diseases, and user-friendly implementation. These challenges may be effectively addressed by combining ML and DL in disease diagnosis. Future research and development initiatives in plant pathology should continue to tackle issues unique to various crops and geographical areas. Through customization of solutions to suit the particular requirements of custard apple, potato, banana, and fig crops, we can guarantee the smooth assimilation of cutting-edge technology into current farming methods. This work establishes the foundation for future efforts to use computational techniques to protect the world's food supply and strengthen the resilience of agricultural systems.

In future work, we plan to validate our model with real-time leaves collected from crop fields and tabulate the accuracy results with the real-time dataset. We will also conduct field studies and consult with agricultural scientists and farmers to assess the model's efficiency. These consultations will provide valuable insights into the practical challenges and possibilities for real-time system deployment, which we will include in our future reports.

## Data Availability

Data derived from public domain resources as: https://doi.org/10.17632/79w2n6b4kf.1https://doi.org/10.17632/jtgh2885yf.2https://doi.org/10.17632/f7dk2yknff.2https://doi.org/10.17632/ptz377bwb8.1

## References

[1] Wang, P., Fan, E. & Wang, P. Comparative analysis of image classification algorithms based on traditional machine learning and deep learning. *Pattern Recogn. Lett.* **141**, 61–67 (2021).

[2] Khan, R. U., Khan, K., Albattah, W. & Qamar, A. M. Image-based detection of plant diseases: From classical machine learning to deep learning journey. *Wirel. Commun. Mob. Comput.* **2021**, 1–13 (2021).35573891

[3] Sujatha, R., Chatterjee, J. M., Jhanjhi, N. Z. & Brohi, S. N. Performance of deep learning vs machine learning in plant leaf disease detection. *Microprocess. Microsyst.* **80**, 103615 (2021).

[4] Ramesh, S., Hebbar, R., Niveditha, M., Pooja, R., Shashank, N., & Vinod, P. V. (2018, April). Plant disease detection using machine learning. In the *2018 International Conference on Design Innovations for 3Cs Compute Communicate Control (ICDI3C)* (pp. 41–45). IEEE.

[5] Atila, Ü., Uçar, M., Akyol, K. & Uçar, E. Plant leaf disease classification using EfficientNet deep learning model. *Eco. Inform.* **61**, 101182 (2021).

[6] Krishnan, V. G., Deepa, J. R. V. P., Rao, P. V., Divya, V. & Kaviarasan, S. An automated segmentation and classification model for banana leaf disease detection. *J. Appl. Biol. Biotechnol.* **10**(1), 213–220 (2022).

[7] Seetharaman, K. & Mahendran, T. Leaf disease detection in banana plant using gabor extraction and region-based convolution neural network. *J. Inst. Eng.(India): SeriesA* **103**(2), 501–507 (2022).

[8] Arman, S. E. *et al.* BananaLSD: A banana leaf images dataset for classification of banana leaf diseases using machine learning. *Data Brief* **50**, 109608 (2023). 37823069 10.1016/j.dib.2023.109608 PMC10562173

[9] Abd Algani, Y. M. *et al.* Leaf disease identification and classification using optimized deep learning. *Measurement Sensors* **25**, 100643 (2023).

[10] Fan, X. *et al.* Leaf image based plant disease identification using transfer learning and feature fusion. *Comput. Electron. Agric.* **196**, 106892 (2022).

[11] Mahum, R. *et al.* A novel framework for potato leaf disease detection using an efficient deep learning model. *Human Ecol. Risk Assess. Int. J.* **29**(2), 303–326 (2023).

[12] Li, L. H., & Tanone, R. (2023, January). Disease Identification in Potato Leaves using Swin Transformer. In *2023 17th International Conference on Ubiquitous Information Management and Communication (IMCOM)* (pp. 1–5). IEEE.

[13] Roy, V. K., Roy, G. K., Thakur, V., Baliyan, N. & Goyal, N. Leaf disease recognition: Comparative analysis of various convolutional neural network algorithms. In *Intelligent Prognostics for Engineering Systems with Machine Learning Techniques* (eds Roy, V. K. *et al.*) (CRC Press, 2024).

[14] Sujatha, R., Mahalakshmi, K. & Chatterjee, J. M. Implementing deep-learning techniques for accurate fruit disease identification. *Plant Pathol.* **72**(9), 1726–1734 (2023).

[15] Trivedi, R. B., Mittal, D., Sahani, A., Fernandes, C. V., Goyal, S., Chatterjee, J. M., & Mehta, V. (2022, June). Predicting the Tomato Plant Disease Using Deep Learning Techniques. In *International Conference on Frontiers of Intelligent Computing: Theory and Applications* (pp. 567–575). Singapore: Springer Nature Singapore.

[16] Gargade, A., & Khandekar, S. (2021). Custard apple leaf parameter analysis, leaf diseases, and nutritional deficiencies detection using machine learning. In *Advances in Signal and Data Processing: Select Proceedings of ICSDP 2019* (pp. 57–74). Springer Singapore.

[17] Al-Ghoul, M. M., Abueleiwa, M. H., Harara, F. E., Okasha, S. & Abu-Naser, S. S. Knowledge based system for diagnosing custard apple diseases and treatment. *Int. J. Acad. Eng. Res.* **6**, 41–45 (2022).

[18] Gargade, A., & Khandekar, S. A. (2019, March). A review: custard apple leaf parameter analysis and leaf disease detection using digital image processing. In *2019 3rd International Conference on Computing Methodologies and Communication (ICCMC)* (pp. 267–271). IEEE.

[19] Meraj, T. *et al.* Computer vision-based plants phenotyping: A comprehensive survey. *Iscience*10.1016/j.isci.2023.108709 (2024). 38269095 10.1016/j.isci.2023.108709 PMC10805646

[20] Mousavi, M., Taskhiri, M. S. & Gandomi, A. H. Standing tree health assessment using contact–ultrasonic testing and machine learning. *Comput. Electron. Agric.* **209**, 107816 (2023).

[21] Kashyap, P. K., Kumar, S., Jaiswal, A., Prasad, M. & Gandomi, A. H. Towards precision agriculture: IoT-enabled intelligent irrigation systems using deep learning neural network. *IEEE Sensors J.* **21**(16), 17479–17491 (2021).

[22] Nagasubramanian, G. *et al.* Ensemble classification and IoT-based pattern recognition for crop disease monitoring system. *IEEE Internet Things J.* **8**(16), 12847–12854 (2021).

[23] Mafi, M. M. H. M. Banana disease recognition dataset. *Mendeley Data*10.1763/79w2n6b4kf.1 (2023).

[24] Thite, S. Sugar Apples / Custard Apples (*Annona squamosa*) disease image dataset. *Mendeley Data*10.17632/jtgh2885yf.2 (2023).

[25] Hafi, S. Fig leaves dataset. *Mendeley Data*10.17632/f7dk2yknff.2 (2023).

[26] Shabrina, N. H. Potato leaf disease dataset in uncontrolled environment. *Mendeley Data*10.17632/ptz377bwb8.1 (2023). 10.1016/j.dib.2023.109955 PMC10733095 38125373

[27] Szegedy, C., Vanhoucke, V., Ioffe, S., Shlens, J., & Wojna, Z. Rethinking the inception architecture for computer vision. In Proceedings of the IEEE conference on computer vision and pattern recognition (pp. 2818–2826). (2016).

[28] Simonyan, K., & Zisserman, A. (2014). Very deep convolutional networks for large-scale image recognition. arXiv preprint arXiv:1409.1556.

[29] Jain, V. & Chatterjee, J. M. *Machine learning with health care perspective* 1–415 (Springer, 2020).

[30] Iwendi, C. *et al.* COVID-19 patient health prediction using boosted random forest algorithm. *Front. public health*10.3389/fpubh.2020.00357 (2020). 32719767 10.3389/fpubh.2020.00357 PMC7350612

[31] Khairandish, M. O., Sharma, M., Jain, V., Chatterjee, J. M. & Jhanjhi, N. Z. A hybrid CNN-SVM threshold segmentation approach for tumor detection and classification of MRI brain images. *Irbm* **43**(4), 290–299 (2022).

[32] Sujatha, R., Chatterjee, J. M., Jhanjhi, N. Z., Tabbakh, T. A. & Almusaylim, Z. A. Heart failure patient survival analysis with multi kernel support vector machine. *Intell. Autom. Soft Comput.*10.32604/iasc.2022.019133 (2022).

[33] Radhakrishnan, S., Lakshminarayanan, A. S., Chatterjee, J. M. & Hemanth, D. J. Forest data visualization and land mapping using support vector machines and decision trees. *Earth Sci Inform.* **13**(1119), 1137 (2020).

[34] Bansal, A. & Jain, A. Utilization of images in an open source software to detect COVID-19. In *Computational Intelligence in Software Modeling* (eds Jain, V. *et al.*) 121–142 (De Gruyter, 2022).

[35] Sagu A, Gill NS, Gulia P, Priyadarshini I, Chatterjee JM. Hybrid Optimization Algorithm for detection of security attacks in IoT-Enabled Cyber-Physical Systems. IEEE Transactions on Big Data. 2024 Jan; 35-46.

[36] Ambarsari, E. W., Awaludin, A. A. R., Suryana, A., Hartuti, P. M. & Rahim, R. Basic concept Pythagoras tree for construct data visualization on decision tree learning. *J. Appl. Eng. Sci.*10.5937/jaes17-21960 (2019).

[37] Wolfram Research, Inc. (n.d.). *Pythagoras Tree -- from Wolfram MathWorld*. https://mathworld.wolfram.com/PythagorasTree.html

[38] Dang-Ngoc, H., Cao, T. N., & Dang-Nguyen, C. (2021, April). Citrus leaf disease detection and classification using hierarchical support vector machine. In *2021 International Symposium on Electrical and Electronics Engineering (ISEE)* (pp. 69–74). IEEE.

[39] Sharma, V., Tripathi, A. K. & Mittal, H. DLMC-Net: Deeper lightweight multi-class classification model for plant leaf disease detection. *Ecol. inform.* **75**, 102025 (2023).

[40] Li, D., Yin, Z., Zhao, Y., Li, J. & Zhang, H. Rehearsal-based class-incremental learning approaches for plant disease classification. *Comput. Electron. Agric.* **224**, 109211 (2024).

